# Echocardiographic Assessment of Left Ventricular Diastolic Function in Adults Between Old and New: Progress and Challenges

**DOI:** 10.3390/diagnostics16020235

**Published:** 2026-01-11

**Authors:** Luca Dell’Angela, Gian Luigi Nicolosi

**Affiliations:** 1 Cardio-Thoracic and Vascular Department, Cardiology Division, Gorizia & Monfalcone Hospital, ASUGI, 34170 Gorizia, Italy; 2 Cardiology, Policlinico San Giorgio, 33170 Pordenone, Italy

**Keywords:** clinical practice, Doppler ultrasound, echocardiography, left ventricular diastolic function, strain echocardiography

## Abstract

Echocardiographic left ventricular (LV) diastolic function assessment represents one of the mainstays for routine, comprehensive transthoracic echocardiography in adults. Estimation of LV filling pressures is an integral part of LV diastolic function evaluation. Additionally, LV diastolic function assessment is crucial for the study of subjects with potential heart failure with preserved LV ejection fraction. Beyond the “old” LV diastolic function parameters, to date, mostly strain-based (and generally artificial intelligence-assisted) additional “new” echocardiographic techniques have emerged to optimize the study of LV diastole. The purpose of the present narrative critical review is to report and discuss the optimal echocardiographic assessment of LV diastolic function in light of the recent literature, with the aim of trying to outline the gaps in the current evidence in view of future developments. To date, multiparametric diastolic evaluation and grading seem advisable, using as many “old and new” measurements as possible—associated with their adequate selection related to the patients’ comorbidities—aiming to cumulatively increase the advantages of diastolic parameters and possibly minimize their limitations. Taking into account the considerable number of echocardiographic measurements to perform and describe, at present, the timing of optimal echocardiography performance and reporting should be adequately adapted to the current technical needs and real-life routine clinical practice. Importantly, contextual clinical and (if needed) multimodality assessment should be included in the diagnostic workflow, in order to enable a more individualized approach.

## 1. Introduction

Echocardiographic left ventricular (LV) diastolic function evaluation represents one of the mainstays for routine, comprehensive transthoracic echocardiographic examination in adults [1,2,3,4,5]. Estimation of LV filling pressures (FPs) is an integral part of LV diastolic function assessment [1,2,3]. Moreover, adequate LV diastolic function evaluation is crucial for the study of patients with potential heart failure (HF) with preserved LV ejection fraction (HFpEF) [1]. Beyond the “standard” LV diastolic function parameters, to date, mostly strain-based echocardiographic techniques and a number of combined parameters have emerged to refine the study of LV diastole [1,6]. Furthermore, the role of artificial intelligence (AI)-assisted echocardiographic modalities is becoming increasingly important in this field, despite a number of limitations [1,7,8,9,10,11,12,13]. The purpose of the present narrative critical review is to report and discuss the optimal echocardiographic assessment of LV diastolic function in light of the recent literature, with the aim of trying to outline the gaps in the current evidence in view of future developments.

## 2. LV Diastolic (Dys)Function: Basic Pathophysiological Considerations and Clinical Implications

**Figure 1:**
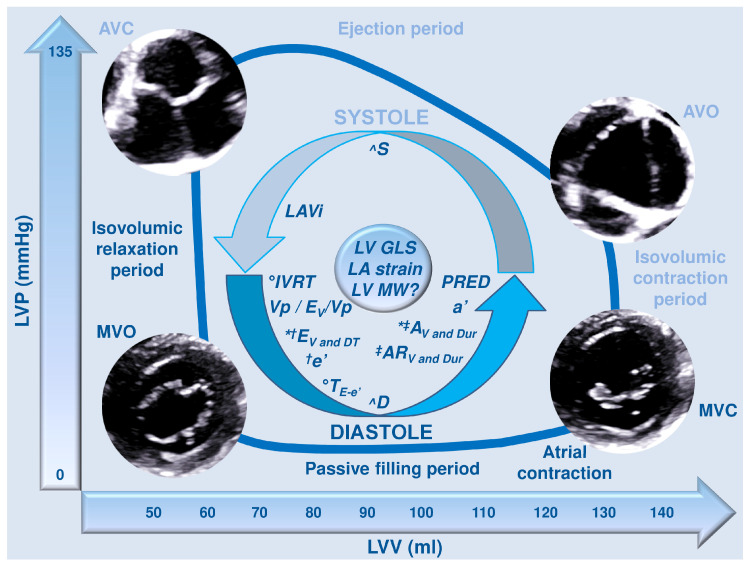
Illustrated explanation of the relationship between cardiac cycle and LV pressure–volume loop, focusing on diastole, as well as main parameters for assessing LV diastolic function. AVC, aortic valve closure; AVO, aortic valve opening; A_V and Dur_, A-wave (peak) velocity and duration; AR_V and Dur_, AR-wave (peak) velocity and duration; D, D-wave (peak) velocity; E_V and DT_, E-wave (peak) velocity and deceleration time; ED, end-diastole/diastolic; GLS, global longitudinal strain; IVRT, isovolumic relaxation time; LA, left atrium/atrial; LAVi, LA volume index; LV, left ventricle/ventricular; LVP, left ventricular pressure; LVV, left ventricular volume; MVC, mitral valve closure; MVO, mitral valve opening; PRED, peak pulmonary regurgitation ED velocity; S, S-wave (peak) velocity; T_E-e’_, TI calculated by subtracting T_E_ (TI between the peak R-wave on ECG and the onset of E-wave) from T_e’_ (TI between the peak R-wave on ECG and the onset of e’-wave), namely, T_e’_—T_E_; TI, time interval; Vp/E_V_/Vp, early diastolic flow propagation velocity (Vp) and E_V_/Vp ratio; ^, and derived S/D ratio; °, and derived IVRT/T_E-e’_ ratio; *, and derived E/A ratio; ^†^, and derived E/e’ ratio; ^‡^, and AR-A duration (see text, tables, and references).

Historically, the definition of diastole is considered tricky and controversial [14]. During the cardiac cycle, the diastole generally includes both a ventricular isovolumic relaxation phase, a passive filling period, and an atrial contraction phase (Figure 1); notwithstanding, diastole might be considered the phase that divides two such consecutive contraction–relaxation transients, namely, including the diastasis and atrial contraction phases [14]. Effectively, some authors [14] have emphasized that a decrease in force and relengthening during myocardial relaxation of an afterloaded contraction may be parts of one activity transient, as well as both a decrease in ventricular pressure and increase in ventricular volume during early rapid filling may be in close relation to such activity transient (thus, considered as part of systole) [14]. Physiologically, heart rate inversely influences the duration of diastole [14]. Diastolic dysfunction causes increased resistance to ventricular filling, an inappropriate upward shift of the diastolic pressure–volume relationship, potential subendocardial ischemia, ventricular remodeling/hypertrophy, and finally leads to symptoms and signs of congestion and HF [1,2,3,14]. Therefore, that pathophysiological process causes both hemodynamic and structural modifications on the complex three-dimensional (3D) organization of the myocardial fibers, evaluable by a number of helpful “old and new” parameters—including Doppler and strain measurements—for adequate characterization, clinical, and prognostic purposes [1,5,14,15,16,17].

## 3. Echocardiographic Evaluation of LV Diastolic Function by Primary Techniques

Primary echocardiographic techniques for evaluating LV diastolic function mainly include Doppler ultrasound parameters, left atrial (LA) volume index (LAVi), and LV global longitudinal strain (GLS) [1,2,3].

### 3.1. Doppler Ultrasound Techniques

**Figure 2:**
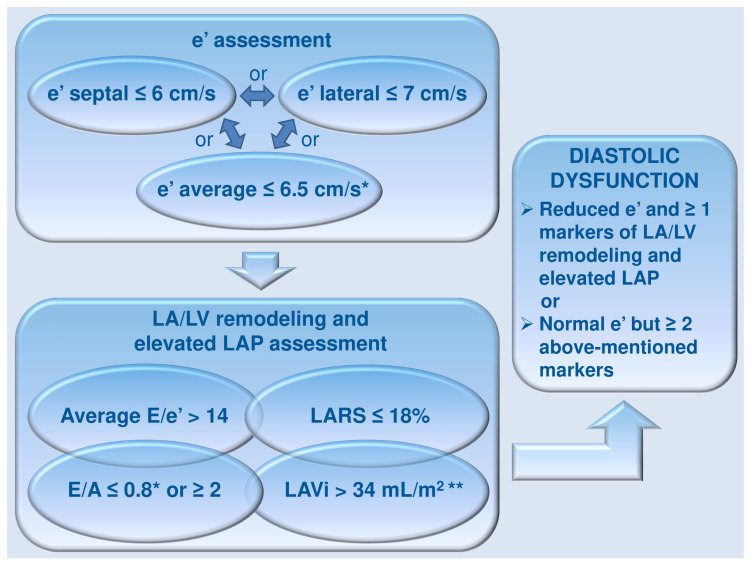
LV diastolic dysfunction diagnostic algorithm. LA, left atrium/atrial; LAP, LA pressure; LARS, LA reservoir strain; LAVi, LA volume index; LV, left ventricle/ventricular; *: mentioned age-specific ranges may be considered for assessing reduced e’ or E/A, after excluding LA dilation in particular conditions, including anemia, AF, AFL, MV disease, or athletes; **: after increased LV mass exclusion in athletes, another finding consistent with diastolic dysfunction is LV mass index >115 g/m^2^ in men or >95 g/m^2^ in women (see text, tables, and references).

**Figure 3:**
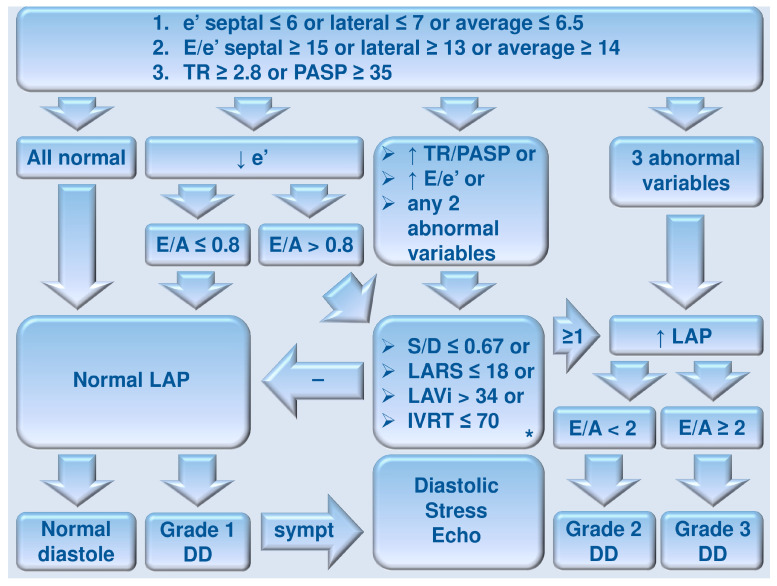
Algorithm of LAP estimation and LV diastolic function grading (exceptions include moderate-to-severe mitral annular calcification, mitral stenosis, severe primary mitral regurgitation, history of mitral valve repair/replacement, history of mitral–transcatheter edge-to-edge repair, atrial fibrillation, history of heart transplantation, LV assist device, non-cardiac pulmonary hypertension, and pericardial constriction). A, A-wave (peak) velocity (measured in m/s); D, peak D-wave velocity (measured in cm/s); DD, diastolic dysfunction; E, peak E-wave velocity (measured in m/s); E/A, E/A (velocity) ratio (adimensional); e’, peak e’-wave velocity (measured in cm/s); E/e’, E/e’ (velocity) ratio (adimensional); IVRT, isovolumic relaxation time (measured in ms); LA, left atrium/atrial; LAP, LA pressure; LARS, LA reservoir strain (peak atrial longitudinal strain); LAVi, LA volume index (measured in mL/m^2^); LV, left ventricle/ventricular; PASP, pulmonary artery systolic pressure (measured in mmHg); S, peak S-wave velocity (measured in cm/s); S/D, S/D (velocity) ratio (adimensional); sympt, if symptom/s; TR, peak tricuspid regurgitation velocity (measured in m/s); *, if none available/reliable, supplemental/secondary methods may be considered, including changes to mitral inflow profile with Valsalva maneuver, L-wave velocity, Ar-A duration, peak pulmonary regurgitation end-diastolic velocity, and pulmonary artery diastolic pressure ≥ 16 mmHg (see text, tables, and references).

**Figure 4:**
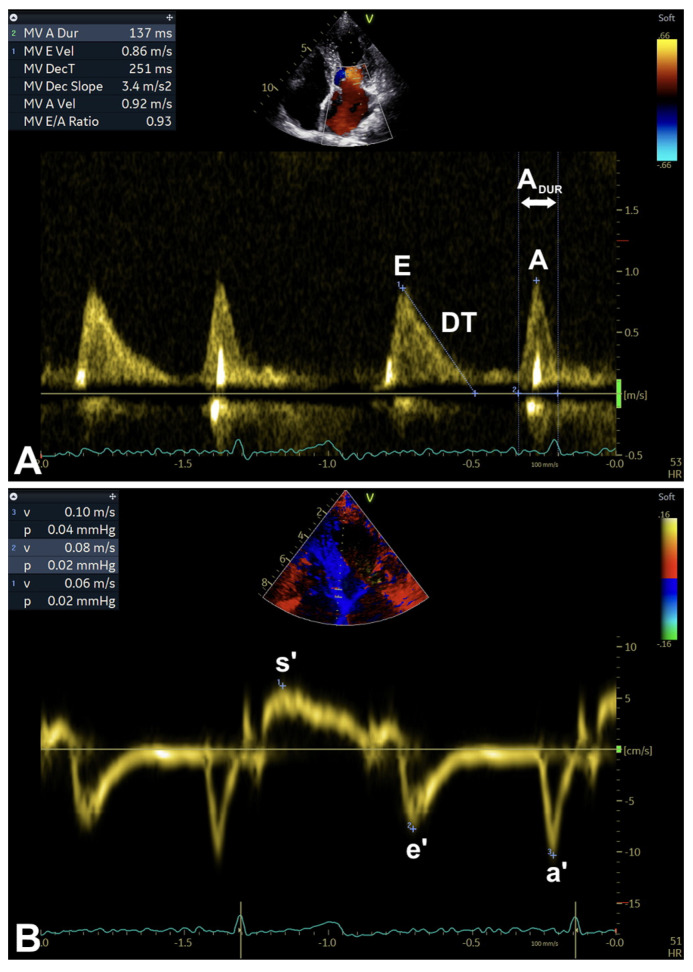
Examples of transmitral inflow (**A**) and TDI (**B**) parameters assessment. A, peak A-wave velocity (after ECG P-wave, peak late diastolic modal velocity; measured in m/s); A_DUR_, A duration (measured in ms); a’, peak a’-wave velocity (after ECG P-wave, peak late diastolic modal velocity; measured in cm/s); DT, deceleration time (measured in ms); E, peak E-wave velocity (after ECG T-wave, peak early diastolic modal velocity; measured in m/s); E/A, E/A (velocity) ratio (adimensional); e’, peak e’-wave velocity (after ECG T-wave, peak early diastolic modal velocity; measured in cm/s); s’, peak s’-wave velocity (at ECG T-wave, peak systolic modal velocity; measured in cm/s); TDI, tissue Doppler imaging. Note: sweep speed at 100 mm/s (see text, tables, and references).

Primary Doppler ultrasound measurements first evaluate transmitral inflow parameters, including E-wave velocity and deceleration time (DT), A-wave velocity and duration, and finally the E/A ratio [1] (Table 1; Figure 1, Figure 2, Figure 3 and Figure 4A). Generally, these parameters are integrated with tissue Doppler imaging (TDI) measurements (e’ velocity and a’ velocity), even in association (such as the E/e’ ratio and average E/e’ ratio), in order to suggest increased LV FPs as well [1] (Table 1; Figure 1, Figure 2 and Figure 4B).

**Figure 5:**
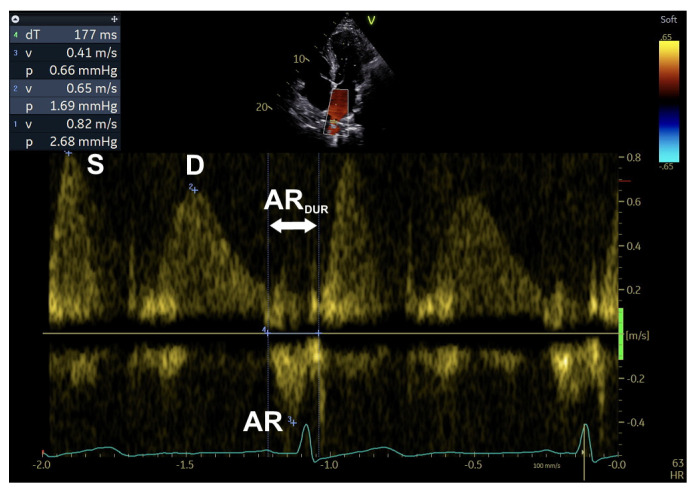
An example of PV inflow parameters assessment. AR, peak AR-wave velocity (after ECG P-wave, peak late diastolic modal velocity; measured in cm/s); AR_DUR_, AR duration (measured in ms); D, peak D-wave velocity (after ECG T-wave, peak early diastolic modal velocity; measured in cm/s); PV, pulmonary veins/venous; S, peak S-wave velocity (at ECG T-wave, peak systolic modal velocity; measured in cm/s); S/D, S/D (velocity) ratio (adimensional). Note: sweep speed at 100 mm/s (see text, tables, and references).

Doppler study on pulmonary venous (PV) inflow provides other useful parameters, such as S-wave velocity, D-wave velocity, S/D ratio, and AR velocity and duration [1] (Table 2; Figure 1 and Figure 5).

**Figure 6:**
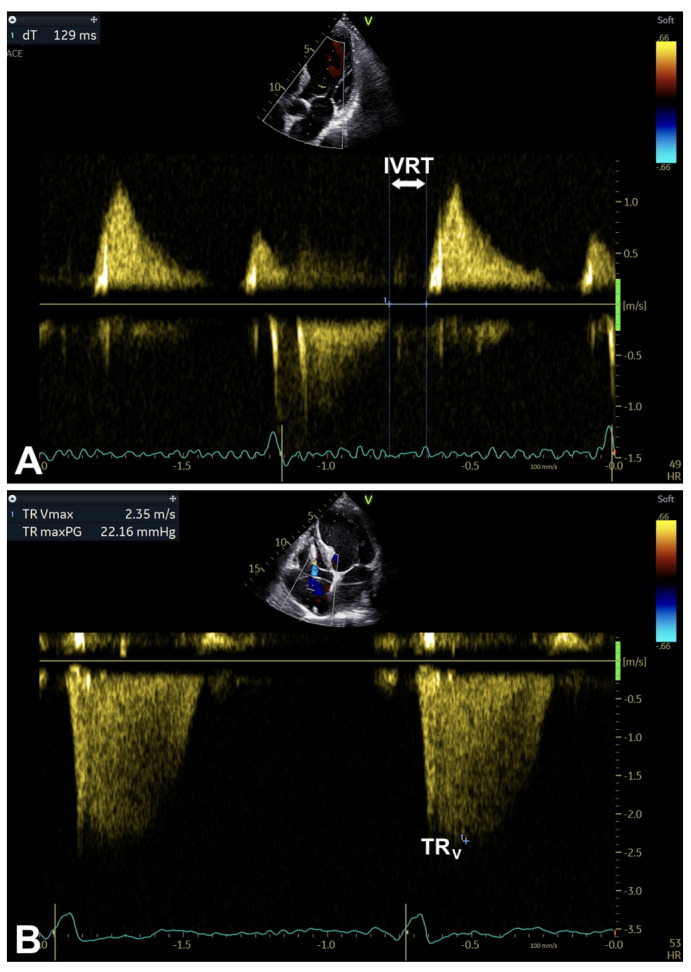
An example of IVRT evaluation (**A**) and CW Doppler study on TR velocity (**B**). These parameters may be helpful for contributing to assess diastolic function and LA pressure. CW, continuous-wave; IVRT, isovolumic relaxation time (measured in ms); TR, tricuspid regurgitation; TR_V_, peak TR velocity. Note: sweep speed at 100 mm/s (see text, tables, and references).

Finally, both isovolumic relaxation time (IVRT) and continuous-wave (CW) Doppler investigation of tricuspid regurgitation (TR) velocity may be helpful in assessing diastolic function and LA pressure [1] (Table 2; Figure 1, Figure 3 and Figure 6A,B).

### 3.2. LA Volume

**Figure 7:**
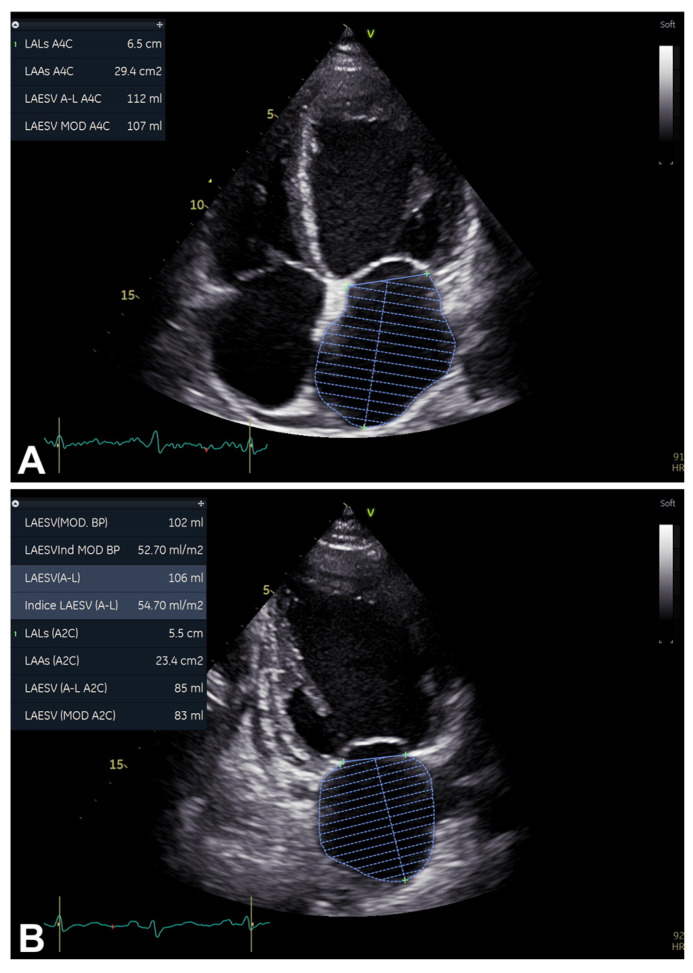
An example of LAVi evaluation, assessed from 2D apical four-chamber (**A**) and two-chamber (**B**) views. Note: 2D, two-dimensional; LAVi, left atrium/atrial volume index (measured in mL/m^2^) (see text, tables, and references).

As mentioned, LAVi is also noted in the primary techniques for assessing LV diastolic function [1] (Table 2; Figure 1, Figure 2, Figure 3 and Figure 7A,B). LAVi is one of the most important criteria for detecting LV diastolic dysfunction and estimating FPs, as well as being a predictor of HF/death, although evidence of a clear prognosis-related threshold is not available to date [1] (Figure 1, Figure 2 and Figure 3). Particularly, a certain degree of physiological LA enlargement is possible, such as in athletes [1,2,3].

Notably, paying attention to avoid any LA image foreshortening, precisely acquiring end-systolic frames [generally, either one or two frame/s before mitral valve (MV) opening], and exactly tracing the LA area are crucial [1] (Table 2; Figure 7A,B).

Despite not being included in the main parameters, the ratio between LA minimal volume (measured at the LV end-diastolic phase) and LV end-diastolic volume (the so-defined “left atrioventricular coupling index”) has been proposed for assessing LV diastolic function—being associated with diastolic dysfunction severity—as well as an independent predictor of outcomes in the presence of HF [18,19,20,21,22]. Notwithstanding, the left atrioventricular coupling index currently seems to perform worse as a prognostic index in the presence of HF with reduced EF in comparison with HFpEF [21]. Moreover, its prognostic role has mainly been demonstrated in selected subjects with stable HF, sinus rhythm, and in the absence of previous valvular interventions, thus further investigations are needed [21].

### 3.3. LV GLS

**Figure 8:**
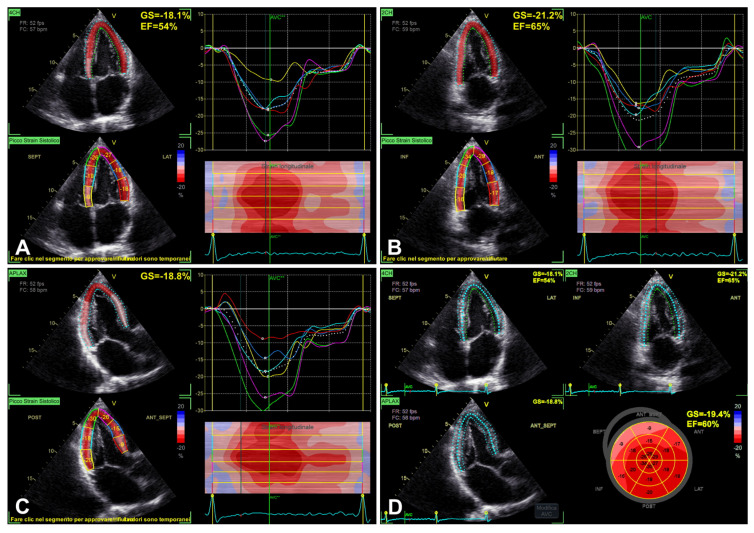
An example of LV GLS evaluation, assessed from 2D apical four-chamber view (**A**), two-chamber view (**B**), three-chamber view (**C**), and reported “bull’s eye” GLS plot (**D**), respectively. Note: 2D, two-dimensional; GLS, global longitudinal strain; LV, left ventricle/ventricular (see text, tables, and references).

Importantly, in light of the recent recommendations, LV GLS represents a “new entry” among primary echocardiographic parameters for evaluating LV diastolic function [1] (Table 2; Figure 1 and Figure 8A–D). Despite LV GLS not being a “direct” index of LV diastolic function, LV GLS has an important “indirect” role [1]. Effectively, reduced LV GLS is present in some patients with diastolic dysfunction associated with normal EF, either with or without HFpEF [1]. Moreover, LV GLS represents an HFpEF evaluation parameter, and is related to worse outcomes in a number of cardiovascular diseases associated with altered diastolic function, including left-sided valvular heart disease and cardiomyopathies [1].

Tran et al. [23] recently enrolled 579 patients in a cross-sectional study, demonstrating that diabetes, concentric LV remodeling/hypertrophy, and LV diastolic dysfunction significantly correlated with abnormal LV GLS (*p*-value < 0.05). Limitations mainly included the cross-sectional study design, only two expert operators involved in the echocardiographic assessment, and some important factors being inadequately addressed, such as length of hypertension and related-conditions management [23].

Although it has the potential property of detecting subtle LV dysfunction—even in the presence of preserved LV EF—, in association with important diagnostic and prognostic implications, a number of limitations should be considered in routine clinical practice [1,6]. LV GLS is possibly influenced by acoustic window (including the role of chest shape), frame rate, preload/afterload, patients’ age, different normality cut-off values, inter-vendor variability, and the literature-mentioned concern associated with the test–retest variability [1,6,24]. Furthermore, the wide overlap of GLS standard deviations between groups at the population level can make the application of a precise physiological meaning to each individual value in each single subject challenging [6].

## 4. Echocardiographic Evaluation of LV Diastolic Function by Secondary Techniques

**Figure 9:**
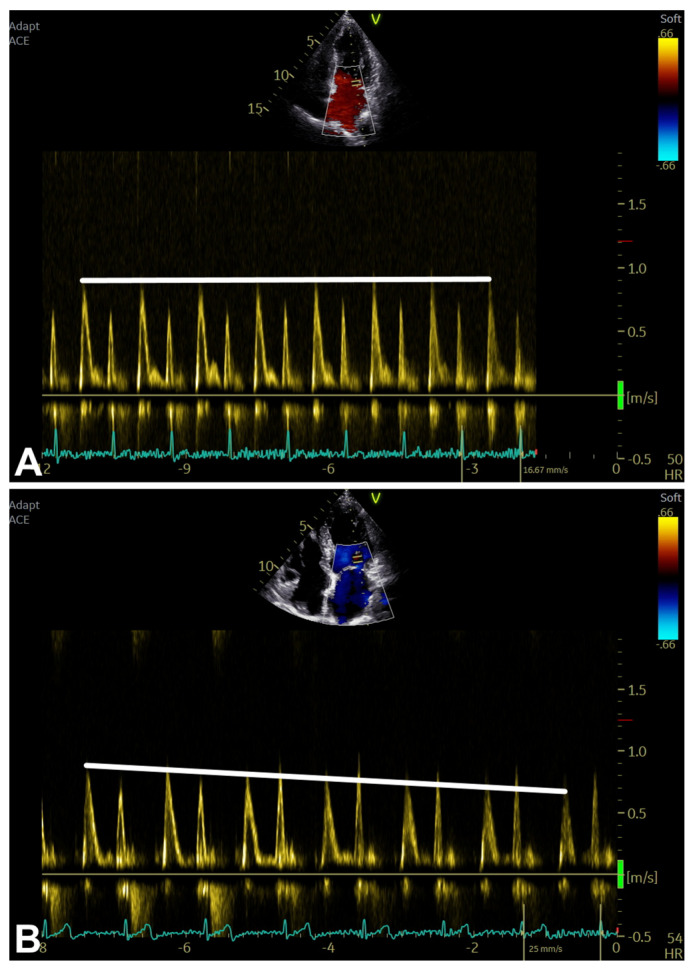
An example of Valsalva maneuver performing in the presence of normal (**A**) and pseudonormal (**B**) transmitral pattern, respectively (see text, tables, and references).

**Figure 10:**
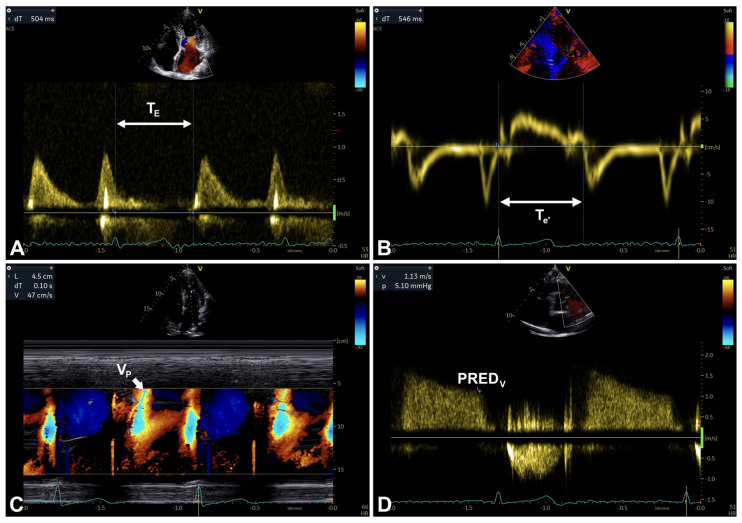
An example of T_E-e’_ time interval [T_E_ (**A**) and T_e’_ (**B**) measurement], Vp (**C**), and PRED_V_^1^ (**D**) assessment. PRED_V_, peak pulmonary regurgitation end-diastolic velocity (measured in m/s); T_E_, TI between ECG peak R-wave and transmitral E-wave onset (measured in ms); T_e’_, TI between ECG peak R-wave and TDI e’-wave onset (measured in ms); T_E-e’_, T_e’_ minus T_E_ (measured in ms); TDI, tissue Doppler imaging; TI, time interval; Vp, color M-mode early-diastolic flow propagation velocity (measured in cm/s) (see text, tables, and references).

Secondary parameters are mainly assessed using Doppler ultrasound techniques, and include the following: Valsalva maneuver-related transmitral inflow analysis (Table 3; Figure 1, Figure 3 and Figure 9A,B), T_E-e’_ time interval (Table 3; Figure 1, Figure 3 and Figure 10A,B), color M-mode early-diastolic flow propagation velocity (Vp) (Table 3; Figure 1, Figure 3 and Figure 10C), and peak pulmonary regurgitation end-diastolic (PRED) velocity [1] (Table 3; Figure 1, Figure 3 and Figure 10D).

Secondary/supplemental parameters are potentially helpful in diagnosing LV diastolic dysfunction [1]; however, they should be considered in a multiparametric evaluation context due to a number of limitations mainly related to their effective feasibility and reproducibility in routine clinical practice [1]. Their adequate selection is crucial, particularly if significant comorbidities are present, potentially making the measurements misleading (e.g., PRED velocity in the presence of pulmonary disease) [1] (Table 3; Figure 1 and Figure 10A–D).

## 5. Advanced Techniques for LV Diastolic Function Assessment

**Figure 11:**
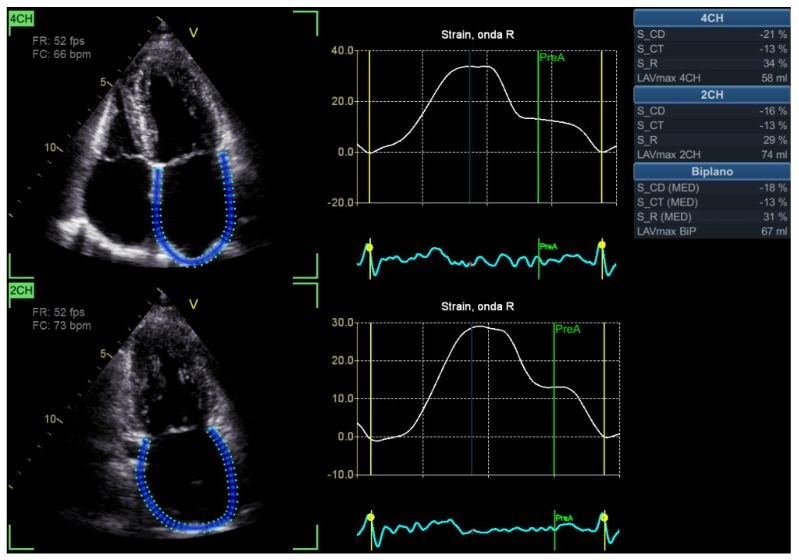
An example of LA function evaluation by LA strain technique, assessed from 2D apical four-chamber (above) and two-chamber (below) views. In light of current recommendations, particularly, biplane LARS represents an important diastolic parameter. Note: 2D, two-dimensional; LA, left atrium/atrial; LARS, LA reservoir strain (peak atrial longitudinal strain) (see text, tables, and references).

**Figure 12:**
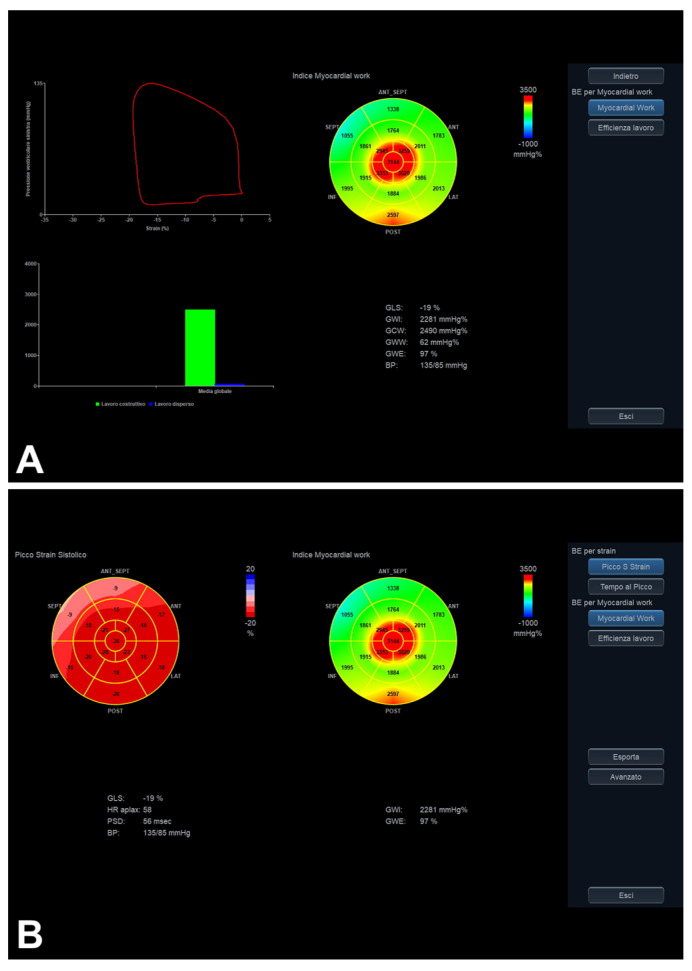
LV function evaluation by MW (**A**) compared with GLS (**B**), in the presence of grade 1 LV diastolic dysfunction and preserved LV EF. In this example, a gradient between basal and apical segments in decreasing longitudinal function is more evident when evaluated by strain-derived MW analysis (whose “bull’s eye” plot looks like an “apical sparing” plot) (**A**), in comparison with GLS (**B**). BP, blood pressure; EF, ejection fraction; GCW, global constructive work; GLS, global longitudinal strain; GWE, global work efficiency; GWI, global work index; GWW, global wasted work; LV, left ventricle/ventricular; MW, myocardial work (see text, tables, and references).

Advanced parameters mainly comprise some strain/strain-derived (and AI-assisted, if available) techniques, including LA strain (Table 4; Figure 1, Figure 2, Figure 3 and Figure 11) and (possibly) LV myocardial work (MW) indices [1,6,16] (Table 4; Figure 1 and Figure 12A,B). Additionally, a new ultrasound-based technique has recently been proposed to detect myocardial stiffness in the presence of diastolic dysfunction—cardiac time-harmonic elastography (THE) [25].

### 5.1. LA Strain

Although not included in the primary/secondary parameters, in light of the recent recommendations, two-dimensional LA strain has become one of the most important criteria for detecting LV diastolic dysfunction [1] (Table 4; Figure 1, Figure 2, Figure 3 and Figure 11). Effectively, LA strain is generally feasible and reproducible, as well as adding important prognostic and predictive value [1].

A number of recent studies have confirmed the added value of two-dimensional LA strain in order to improve diastole-related risk stratification, particularly in the presence of preserved LV EF and sinus rhythm [26,27,28]. Mainly, LA reservoir strain (LARS) enhanced risk stratification [26,27,28], and the ratio of E/e’ and LARS has been proposed to measure LA stiffness and better characterize LA performance [29].

Nonetheless, a number of limitations should be considered [1]. Particularly, some concerns associated with age/load dependency, arrhythmias/bundle branch block (R-R gating may be inaccurate), particular anatomical conditions (e.g., mobile atrial septum, thin-walled left atrium, and mitral annular calcification), significant mitral regurgitation, and history of heart transplantation have been pointed out [1]. Moreover, adequate operator skills, a good ultrasound window (chest-shape dependency should be considered), inter-vendor/inter-software variability, and dedicated software package availability must be mentioned [1,6,24].

In a single-center study enrolling both 72 subjects with metabolic syndrome and 34 healthy volunteers, four-dimensional LA strain has been mentioned for successfully contributing to diastolic function evaluation [30]. To note, a number of other limitations has been pointed out, including the lack of specific threshold values for definition/validation in association with the LV diastolic dysfunction group, as well as the unclear relationship between LA functional changes and disease progression/medication administration [30]. In addition, as the ratio of 3D-measured LA volume index to TDI-derived a’ velocity at the median mitral annulus, LA volumetric/mechanical coupling index (LACI) has recently been proposed for assessing LA performance in this field [31,32]. Notably, many of the above-mentioned limitations remain, also comprehending its challenging usefulness associated with load dependency (LACI includes an LA booster function, thus LACI is influenced by conduit function as well), arrhythmias (e.g., atrial fibrillation and/or absence of a regular rhythm), atrio-ventricular geometry changes that may affect LACI measurements, and possible pulmonary hypertension-related factors [31,32].

### 5.2. LV MW

LV MW is a relatively recent GLS-derived parameter for assessing LV function [6,16,33]. By adding arterial pressure measurement to GLS, LV MW is born to reduce the (after)load-dependency limitations of LV GLS as well [16,33]. Although not yet introduced in current diastolic assessment guidelines [1], in light of the recent literature, the potential added value of LV MW has been mentioned in this field [34,35,36] (Table 4; Figure 1 and Figure 12A,B).

As for LV GLS [1], taking into account the above-mentioned considerations, the potential MW contribution may be “indirect” for assessing LV diastolic function. Chilingaryan et al. [34] recently studied 215 subjects (5 patients were excluded due to myocardial infarction; mean age, 73 ± 8 years; 63% women; follow-up, 3 years; two experienced echocardiographers performed the echocardiographic exams) with HFpEF and preclinical diastolic dysfunction. The authors [34] showed that LV global constructive work (GCW) predicts EF decline (not related to baseline EF values) in those patients, possibly because GCW reflects LV endocardial constructive shortening in association with afterload. A recent prospective case-control study [35] assessed 133 women receiving anthracycline-based chemotherapy compared to a control group of 65 age-matched healthy women (mean age, 52 ± 12 and 51 ± 6 years, respectively; *p*-value = 0.263). The chemotherapy group was subsequently subdivided into two groups, namely a chemotherapy-related cardiac dysfunction group and a chemotherapy-related cardiac dysfunction-free group, including 37 and 88 women, respectively [35]. In particular, in the logistic regression analysis, the global work index (GWI) (*p*-value < 0.001) and GCW (*p*-value = 0.003) were shown as significant predictors of chemotherapy-related cardiac dysfunction, as well as diastolic stress echocardiography-related peak TR velocity (*p*-value < 0.001), Troponin (*p*-value < 0.001), NT-proBNP (*p*-value < 0.001), LV EF (*p*-value = 0.021), and GLS (*p*-value < 0.001) [35]. Notably, several limitations were pointed out, including the single-center design, the small number of subjects developing chemotherapy-related cardiac dysfunction (limiting power for statistical analyses), the short 12-month follow-up duration, subjects with a suboptimal acoustic window (e.g., due to obesity or breast surgery) were excluded, and, importantly, a single experienced operator performed the GLS/MW analyses and diastolic stress echocardiography (not reflecting real-world/multicenter conditions) [35]. Moreover, Jasaityte et al. [36] included 224 women (115 normotensive women versus 69 women with arterial hypertension) in their study, aiming to analyze determinants of MW indices in association with diastolic dysfunction as well; both arterial hypertension and GLS were found to be significantly related to GWI and GCW [36].

Generally, the main limitations of LV MW include the many GLS-related limits, non-invasive arterial pressure measurement-related concerns, and the single available software [6,16,33]. Despite the mentioned GLS and MW intra- and inter-operator reproducibility in the literature [1,15,16], both the GLS and MW analyses were generally and systematically performed by a few expert operators to date [34,35] and, therefore, do not reflect real-world routine clinical practice [6,33].

### 5.3. Cardiac THE

A new ultrasound-based tool seems available for detecting myocardial stiffness in the presence of diastolic dysfunction—cardiac THE [25]. Enabling the detection and mapping of increased diastolic myocardial stiffness is the main advantage of cardiac THE [25]. Employing both standard medical ultrasound and continuous external vibration for regionally resolved mapping of diastolic shear wave (as a myocardial stiffness indicator), Meyer et al. [25] prospectively selected and studied 109 patients, divided into three groups: 54 healthy controls (47 ± 16 years; 28 men and 26 women; controls versus patients untreated with tafamidis, *p*-value < 0.001; controls versus patients treated with tafamidis, *p*-value < 0.001); 10 subjects with moderate LV hypertrophy (70 ± 14 years; five men and five women; controls versus patients with moderate LV hypertrophy, *p*-value < 0.001; patients with moderate LV hypertrophy versus untreated with tafamidis, *p*-value = 0.056; patients with moderate LV hypertrophy versus treated with tafamidis, *p*-value = 0.044); and 45 subjects with wild-type transthyretin amyloidosis (41 men and 4 women), subdivided in 20 patients treated with tafamidis (81 ± 7 years) and 35 untreated subjects (80 ± 7 years; patients untreated versus treated with tafamidis, *p*-value = 0.696). That single-center prospective study [25] showed cardiac THE as a cost-effective technique, allowing for abnormal myocardial stiffness detection in the presence of diastolic dysfunction. The above-mentioned non-invasive ultrasound-based modality also seemed accurate at greater depths of up to 15 cm, independent of both region selection and body mass index [25]. The limitations include small group sizes, technical limits associated with current restrictions to two-dimensional acquisition/evaluation of diastolic phases only, and THE setup relying on a vibrating bed that is not easily movable to the subject [25].

## 6. Gaps in the Evidence and Future Perspectives on LV Diastolic Function Assessment

Echocardiographic LV diastolic function and FPs assessment are crucial for routine, comprehensive transthoracic echocardiography in adults in order to evaluate subjects with potential HFpEF as well [1,2,3]. Beyond the “old” LV diastolic function measurements, additional “new” echocardiographic parameters have emerged for optimizing the study of LV diastole [1,37].

Some “old” LV diastolic function parameters remain among the so-called primary techniques, including PW Doppler transmitral inflow and tissue parameters, PW Doppler study on PV inflow, CW Doppler study on TR velocity and IVRT, as well as LAVi [1].

Considering a number of concerns regarding the feasibility and reproducibility, as well as potential comorbidities that may generate misleading results, Valsalva maneuver-related transmitral inflow analysis, T_E-e’_ time interval, Vp, and PRED velocity are included in the secondary/supplemental parameters [1].

Importantly, in light of recent recommendations, LV GLS actively takes part in the diagnostic echocardiographic workflow [1,37]. Likewise, taking into account the mentioned GLS considerations, LV MW might be a further parameter that can contribute to diagnoses [34,35,36]. Notably, although “reassuring” considerations of GLS and MW intra- and inter-operator reproducibility are mentioned in the literature [1,15,16], both GLS and MW analyses were generally and systematically performed by only a few expert operators to date [34,35], and, therefore, do not reflect real-world routine clinical practice [6,33]. On this topic, real-world multicenter research should be considered for the future [6,33].

Despite not being included in the primary/secondary techniques, in light of recent recommendations, two-dimensional LARS has become one of the most important parameters for LV diastolic dysfunction assessment, enhancing risk stratification as well [1,26,27,28,37]. Also, the ratio of E/e’ and LARS has been proposed to measure LA stiffness and better characterize LA performance [29]. Research on possible promising four-dimensional LA strain is ongoing [30].

Interestingly, besides the ratio of E/e’ and LARS, other parameters seem promising in further refining comprehensive diastolic evaluation and characterization [18,19,20,21,22,31,32]. Effectively, the ratio between LA minimal volume and LV end-diastolic volume (“left atrioventricular coupling index”) may help assess LV diastolic function, being associated with diastolic dysfunction severity and an independent predictor of outcomes in the presence of HF [18,19,20,21,22]. Notwithstanding, left atrioventricular coupling index currently seems to perform worse in the presence of HF with reduced EF, and its prognostic role has mainly been demonstrated in selected subjects with stable HF, sinus rhythm, and in the absence of previous valvular interventions [21]. Additionally, the ratio of 3D-measured LA volume index to TDI-derived a’ velocity at the median mitral annulus (LACI) seems to assess LA performance [31,32]. However, some limitations have been highlighted, noting its challenging usefulness associated with load dependency, arrhythmias (potentially misleading in the absence of a regular rhythm), possible atrio-ventricular geometry changes, and pulmonary hypertension-related concerns [31,32].

Furthermore, cardiac THE represents a potential diagnostic tool for detecting myocardial stiffness in the presence of diastolic dysfunction [25]. Notwithstanding, further studies are needed to better explore this ultrasound-based modality [25].

To date, echocardiographic multiparametric diastolic evaluation seems advisable, employing as many “old and new” measurements as possible—associated with their adequate selection related to the patients’ comorbidities—in order to increase the advantages of the diastolic parameters and possibly minimize their limitations [1]. In particular, with respect to strain and strain-derived techniques, the wide overlap of standard deviations between groups at the population level can make the application of a precise physiological meaning to each individual value in each single subject challenging [6,33]. Moreover, the role of AI-assisted echocardiographic modalities is becoming increasingly important in this field as well, despite a number of persisting AI-related concerns, including interpretability/transparency of AI algorithms and the still-important need for operator supervision of AI-assisted measurements [1,6,7,8,9,10,11,12,13,33].

Taking into account the current considerable number of echocardiographic measurements to perform and describe in the report, the timing of optimal echocardiography performance and reporting should be adequately adapted to the actual technical needs and real-life routine clinical practice [38,39,40]. In order to speed up final reporting, it is possible that AI-assisted dedicated software may be considered in the future [38,39,40].

Importantly, contextual clinical and possible multimodality assessments—including both biomarkers (such as natriuretic peptides) and additional instrumental tools (e.g., cardiac magnetic resonance, nuclear imaging, and invasive hemodynamic evaluation)—should be included in the diagnostic workflow when indicated, in order to enable a more individualized approach [1,37].

## 7. Conclusions

For accurate diastolic function assessment, to date, many (possibly AI-assisted) measurements have solid evidence of both diagnostic and prognostic efficacy, while other parameters require more evidence and further developments [1,2,3,5,6,8,9,10,11,12,13,15,16,17,23,25,30,37].

The “old” (mainly) Doppler ultrasound-based parameters mostly require both the optimal collaboration of the patient/s and the absence of significant comorbidities that potentially make some parameters misleading (e.g., PRED velocity in the presence of pulmonary disease) [1].

The “new” (mainly) strain-based parameters also require a higher ultrasound window quality, and are more challenging in the presence of some anatomical conditions (e.g., mobile atrial septum, thin-walled left atrium, mitral annular calcification, and particular chest shapes) [1,6,24,33]. Moreover, other factors should be considered, including the inter-vendor/inter-software variability, dedicated software package availability, as well as greater difficulty in performing strain-based measurements in the presence of a larger spectrum of arrhythmias [1,6,33].

In conclusion, echocardiographic multiparametric diastolic evaluation and grading is advisable, using as many “old and new” measurements as possible—associated with their adequate selection related to the patients’ comorbidities—aiming to cumulatively increase the advantages of diastolic parameters and possibly minimize their limitations [1,2,3,6,33]. Taking into account the current considerable number of echocardiographic measurements to perform and describe, the timing of optimal echocardiography performance and reporting should be adequately adapted to the actual technical needs and real-life routine clinical practice, and AI-assisted dedicated software may be considered in next future in order to speed up the final reporting [38,39,40]. Notably, contextual clinical and (if needed) multimodality assessment should be included in the diagnostic workflow, aiming to enable a more individualized approach [1,37].

## Figures and Tables

**Table 1:** Main primary echocardiographic parameters for assessing LV diastolic function: transmitral inflow and TDI at mitral annulus [1,2,3,17].

Parameter/Variable	Acquisition	Measurements/Pathophysiological Considerations	Advantages	Limitations
**Transmitral inflow**	- Apical 4C view, color and spectral (PW) Doppler imaging. - Preferably, low wall filter setting (100–200 MHz), low signal gain, PW Doppler SV 1–3 mm, and sweep speed at 100 mm/s are needed.	**Peak E-wave velocity (or E)** (measured in m/s): After ECG T-wave, measure peak early diastolic modal velocity; During early diastole, indicates the LA-to-LV pressure gradient; Affected by LV relaxation and LAP rate modifications. Range (20–39 y): 0.54 (0.52–0.57) to 1.11 (1.07–1.16); Range (40–60 y): 0.47 (0.46–0.49) to 1.02 (0.99–1.05); Range (60–80 y): 0.39 (0.37–0.42) to 0.92 (0.88–0.96).	**- Feasible.** **- Reproducible.** **- Diagnostic and prognostic information** (e.g., peak E-wave velocity and E/A ratio: predictor of clinical outcome in subjects with DCM and reduced LV EF; short DT in reduced LV EF reflects increased LV EDP; in the presence of cardiac disease, short A-wave duration reflects high LV FP).	**- Related to age** (e.g., both peak E-wave velocity and E/A decrease with age; both DT and peak A-wave velocity increase with age). **- Related to preload.** **- US beam optimal alignment-related.** **- Challenging in the presence of arrhythmias** (e.g., both peak/duration of A-wave velocity and E/A not applicable in AF/AFL; DT not applicable in AFL; peak A-wave velocity possibly not feasible if paced rhythm, sinus tachycardia, and first-degree AV block; A-wave duration not feasible if E-A fusion, sinus arrhythmias, second- and third-degree AV block, and ECG PR interval < 120 ms). - Other: peak E-wave velocity poorly correlates with LV FP in subjects with CAD and/or HCM with LV EF >50%.
		**DT** (in ms): Along the deceleration slope, measure TI from peak E-wave velocity to the zero baseline; May be used to determine filling patterns (normal, IR, PN, and RF). Range: in the presence of reduced LV EF, if <140 ms reflects increased LV EDP (high accuracy in both SR and AF).		
		**Peak A-wave velocity (or A)** (in m/s): After ECG P-wave, measure peak late diastolic modal velocity; During late diastole, indicates the LA-to-LV pressure gradient; Affected by LV compliance and LA contractile function. Range (20–39 y): 0.24 (0.21–0.27) to 0.68 (0.63–0.72); Range (40–60 y): 0.33 (0.32–0.35) to 0.82 (0.80–0.84); Range (60–80 y): 0.43 (0.40–0.45) to 0.97 (0.93–1.00).		
		**E/A ratio (or E/A)** (adimensional): Peak E-wave velocity divided by peak A-wave velocity; May be used to determine filling patterns (normal, IR, PN, and RF). Range (20–39 y): 0.88 (0.82–0.94) to 2.73 (2.66–2.81); Range (40–60 y): 0.69 (0.66–0.73) to 2.07 (2.03–2.11); Range (60–80 y): 0.50 (0.45–0.56) to 1.40 (1.34–1.47).		
		**A-wave duration** (in ms): At zero baseline, measure TI from the onset to the offset of the A-wave signal; During late diastole, indicates LV compliance; Particularly helpful if used with pulmonary venous AR duration. Range: in the presence of cardiac disease, if <120 ms reflects increased LV FP.		
		**L-wave velocity (or L)** (in m/s): In the presence of triphasic transmitral inflow pattern, represents the mid-diastolic flow; During diastasis, indicates the continued LA-to-LV pressure gradient associated with delayed LV relaxation, as well as high LV FP probably; In the presence of LVH/HCM, is specific for high LV FP (but low sensitivity); in the presence of AF, may be related to high LV FP. Range: if (rarely) present in subjects with normal LV diastolic function and bradycardia, it is usually <0.4.		
**TDI** **(at mitral annulus)**	- Apical 4C view with TDI preset optimization *. - Alignment optimization (angle of interrogation should be parallel to annular motion). - Preferably, PW Doppler SV 5–10 mm and sweep speed at 100 mm/s are needed.	**Peak e’-wave velocity (or e’)** (measured in cm/s): After ECG T-wave, measure peak early diastolic modal velocity; Index of LV relaxation and less load-dependent if compared to other conventional PW Doppler parameters; Affected by LV relaxation, restoring forces and FP; Helpful for differentiation PN from normal transmitral inflow patterns. ***- Lateral e’***: Range (20–39 y): 9.9 (9.4–10.4) to 22.1 (21.5–22.8); Range (40–60 y): 7.5 (7.3–7.8) to 17.5 (17.1–17.9); Range (60–80 y): 5.2 (4.8–5.6) to 13.0 (12.4–13.5). ***- Septal e’***: Range (20–39 y): 7.2 (6.8–7.7) to 16.4 (16.0–16.9); Range (40–60 y): 5.7 (5.4–5.9) to 13.5 (13.2–13.8); Range (60–80 y): 4.1 (3.7–4.5) to 10.6 (10.1–11.0). ***- Average e’***: Range (20–39 y): 8.7 (8.2–9.2) to 19.1 (18.6–19.7); Range (40–60 y): 6.7 (6.4–7.0) to 15.4 (15.1–15.7); Range (60–80 y): 4.7 (4.3–5.1) to 11.7 (11.2–12.2).	**- Feasible.** **- Reproducible.** **- Diagnostic and prognostic information.**	**- Related to age** (e.g., peak e’-wave velocity decreases with age). **- US beam optimal alignment-related.** **- Challenging in the presence of arrhythmias.** **- Significantly limited accuracy in some conditions** (e.g., relevant MAC, prosthetic mitral ring/valve, pericardial disease, CAD in the presence of regional dysfunction in the sampled segments).
		**Peak a’-wave velocity (or a’)** (in cm/s): after ECG P-wave, measure peak late diastolic modal velocity.		
		**MV E/TDI e’ ratio (or E/e’)** (adimensional): MV peak E-wave velocity (in cm/s) divided by TDI peak e’-wave velocity (in cm/s); Helpful for predicting increased LV FP. Range: generally, if <8 usually indicates normal LV FP, and if >14 usually indicates increased LV FP with high specificity. ***- Lateral E/e’***: Range (20–39 y): 2.5 (2.0–3.0) to 6.3 (5.3–7.2); Range (40–60 y): 3.6 (3.4–3.9) to 9.4 (8.9–10.0); Range (60–80 y): 4.8 (4.5–5.0) to 12.6 (12.0–13.2). ***- Septal E/e’***: Range (20–39 y): 4.0 (3.3–4.7) to 9.1 (8.2–9.9); Range (40–60 y): 4.9 (4.6–5.3) to 12.1 (11.7–12.6); Range (60–80 y): 5.9 (5.5–6.3) to 15.2 (14.7–15.7). ***- Average E/e’***: Range (20–39 y): 4.0 (3.8–4.3) to 9.1 (8.5–9.7); Range (40–60 y):4.6 (4.4–4.8) to 11.5 (11.2–11.9); Range (60–80 y): 5.2 (4.9–5.4) to 14.0 (13.4–14.5).		
		**MV E/Average TDI e’ ratio (or Average E/e’)** (adimensional): MV peak E-wave velocity (in cm/s) divided by the average of TDI septal peak e’-wave velocity (in cm/s) and TDI lateral e’-wave velocity (in cm/s).		

Legend: 4C, four-chamber; AF, atrial fibrillation; AFL, atrial flutter; AR, atrial reversal; CAD, coronary artery disease; DT, deceleration time; ECG, electrocardiogram; EDP, end-diastolic pressure; EF, ejection fraction; FP, filling pressure/s; HCM, hypertrophic cardiomyopathy; IR, impaired relaxation; LA, left atrium/atrial; LAP, left atrial pressure; LV, left ventricle/ventricular; LVH, left ventricular hypertrophy; MAC, mitral annular calcification; MV, mitral valve; NR, normal range; PN, pseudonormal; PW, pulsed-wave; RF, restrictive filling; SR, sinus rhythm; SV, sample volume; TDI, tissue Doppler imaging; TI, time interval; US, ultrasound; y, year/s; *, low-velocity detection with high-amplitude signals, PW sample volume of 5–10 mm (at septal and lateral insertion site of the mitral leaflets); reference values displayed are 5th percentile limit to 95th percentile limit (with 95% confidence limits) (see text and references).

**Table 2:** Main primary echocardiographic parameters for assessing LV diastolic function: PV inflow, TR, IVRT, LAVi, and LV GLS [1,2,3,6].

Parameter/Variable	Acquisition	Measurements/Pathophysiological Considerations	Advantages	Limitations
**PV inflow**	- Apical 4C view with color/PW Doppler imaging (and reduced NL: high PRF may be needed when peak velocities exceed the NL). - PW Doppler SV (3–5 mm) placed 5–10 mm (generally) into the right upper and/or right lower PV. - Preferably, low wall filter setting (100–200 MHz), low signal gain, and sweep speed at 100 mm/s are needed.	**Peak S-wave velocity (or S)** (measured in cm/s): At ECG T-wave, measure peak systolic modal velocity (in the presence of two systolic peaks S1 and S2, S2 should be considered for S/D ratio); Affected by LAP, LA contractility/relaxation/stiffness, and LV/RV contractility. Range: in the presence of S/D ratio < 1, SFF < 40%, and reduced LV EF, reduced S indicates high LAP.	**- Generally feasible.** **- Diagnostic and prognostic information.**	**- Possibly suboptimal in ICU subjects.** **- (S, D, and S/D) in the presence of preserved LV EF, MV disease, HCM, and AF, less accurate relationship between PV systolic filling fraction and LAP.** **- Challenging in the presence of arrhythmias** (e.g., in the presence of AF, AR velocity is absent; in the presence of heart block, sinus tachycardia, and atrial arrhythmias, Ar-A is not applicable).
		**Peak D-wave velocity (or D)** (in cm/s): After ECG T-wave, measure peak early diastolic modal velocity; Affected by both LV relaxation and LAP in early diastole; Changes in parallel with E. Range: in the presence of AF, DT of D-wave velocity ≤ 220 ms indicates high LV FP.		
		**S/D ratio (or S/D)** (adimensional): Peak S-wave velocity divided by peak D-wave velocity; May be used to determine filling patterns (normal, IR, PN, and RF); Inversely related to LAP. Range: presence of S/D ratio < 1, SFF < 40%, reduced LV EF, and reduced S suggest high LAP.		
		**Peak AR-wave velocity** (in cm/s): After ECG P-wave, measure peak late diastolic modal velocity; Affected by both LV compliance and LA contractility; Helpful when elevated LV EDP and normal LAP have been suspected (e.g., grade 1 diastolic dysfunction). Range: if >35 cm/s, indicates an increased LV EDP.		
		**AR-wave duration** (in ms): At zero baseline, measure TI from the onset to the offset of the AR-wave signal; Longer AR duration, increased AR and D velocities, and decreased S velocity are related to both decreased LV compliance and increased LAP.		
		**AR-A duration (or Ar-A)** (in ms): Time difference between AR-wave and A-wave durations; Age- and LV EF-independent; Related to LV EDP/FP. Range: if ≥30 ms, indicates increased LV EDP/FP (particularly, accurate in the presence of HCM and MR).		
**TR**	- From any view that allows correct alignment of US beam in parallel with TR jet (CW Doppler). - Preferably, sweep speed at 50–100 mm/s is needed.	**Peak TR velocity** (measured in m/s): Averaged over the respiratory cycle; In the absence of PS/RVOT obstruction, estimates the PASP (in the absence of pulmonary disease, high PASP suggests high LAP). Ranges, (20–39 y): 1.3 (1.1–1.5) to 2.7 (2.6–2.7); (40–60 y): 1.5 (1.4–1.6) to 2.7 (2.7–2.7); (60–80 y): 1.7 (1.5–1.8) to 2.8 (2.7–2.8).	**- Generally feasible and reproducible.** **- Diagnostic and prognostic information.**	**- Indirect LAP estimation.** **- Related to age.** **- US beam optimal alignment-related.** **- Significantly limitations in some conditions** (e.g., PS, RVOT obstruction, very severe TR and low systolic RV-RA pressure gradient).
**IVRT**	- Apical 3C (or long-axis) or 5C view with color/CW Doppler imaging (CW Doppler through LVOT in order to simultaneously display the end of aortic ejection and transmitral inflow onset). - Preferably, low wall filter setting (100–200 MHz), low signal gain, and sweep speed at 100 mm/s are needed.	**IVRT duration (or IVRT)** (measured in ms): At zero baseline, measure TI between AVC and MVO; Determines the TI between AVC and MVO, as well as the crossover between LA and LV pressures; Directly related to LV relaxation and inversely related to LAP; Useful for estimating LV FP (e.g., in the presence of MAC; in the presence of HFrEF, in combination with transmitral inflow measurements; in the presence of MS/MR, in combination with T_E-e’_). Range: LAP likely normal if >110 ms; if <70 ms, high specificity for elevated LAP in the presence of cardiac disease.	**- Generally feasible and reproducible.**	**- More helpful in combination with other parameters.** **- Related to preload** (tends to normalize with increasing LAP). **- Partially affected by HR and AP.** **- Related to age** (shorter in young subjects, lengthens with age).
**LAVi**	- Apical 2C and 4C views. - Maximize both LA base width and LA long axis.	**LAVi** (measured in mL/mq): From each view, acquire ES frames and trace LA area (excluding PV and LAA), thus (generally automatic) LAV calculation using the method of disks or area-length method (subsequently divided by BSA); Reflects the increased LV FP effects; LA dilation is an independent predictor of death, HF, AF and ischemic stroke. ***- LAVi***: Range (20–39 y): 12.1 (10.9–13.2) to 39.4 (34.6–44.2); Range (40–60 y): 12.9 (12.2–13.5) to 38.3 (35.4–41.1); Range (60–80 y): 13.7 (12.7–14.6) to 37.1 (33.0–41.3). ***- LAVi (Simpson’s method)***: Range (20–39 y): 12.5 (12.0–13.0) to 41.9 (38.1–45.6); Range (40–60 y): 13.3 (13.0–13.6) to 41.0 (38.5–43.4); Range (60–80 y): 14.2 (13.7–14.6) to 40.0 (36.5–43.6). ***- LAVi (A-L)***: Range (20–39 y): 8.9 (3.9–13.9) to 20.9 (12.9–28.8); Range (40–60 y): 11.0 (8.9–13.0) to 27.1 (24.0–30.3); Range (60–80 y): 13.0 (9.9–16.0) to 33.4 (28.6–38.2).	**- Feasible and reproducible.** **- Diagnostic and prognostic information.**	**- Good image quality is needed.** **- Related to age**.
**LV GLS**	- 2D apical 4C, 3C, and 2C views. - Optimize 2D gains, FR (40–80 frames/s), ECG. - Acquire 3-to-5 cardiac cycles for each view to obtain similar HRs.	**LV GLS** (measured in %): Probe in apical zone with 4C, 3C, and 2C views; Dedicated (AI-assisted, if available) strain software automatically tracks LV endocardium and calculates LV GLS; LV systolic function measurement (with strain-derived EF).	**- Generally feasible and reproducible** (mainly achievable with dedicated software package, adequate operator skills, and a good US window). **- Prognostic and predictive value.**	**- Age- and load-dependent.** **- Chest-shape-dependent.** **- Image quality-related.** **- Inter-vendor and inter-software variability.**

Legend: 2C, two-chamber; 2D, two-dimensional; 3C, three-chamber; 4C, four-chamber; 5C, five-chamber; AF, atrial fibrillation; AFL, atrial flutter; AVC, aortic valve closure; BSA, body surface area; CW, continuous-wave; DT, deceleration time; ECG, electrocardiogram; EDP, end-diastolic pressure; EF, ejection fraction; ES, end-systolic; FP, filling pressure/s; FR, frame rate; GLS, global longitudinal strain; HCM, hypertrophic cardiomyopathy; HF, heart failure; HR/s, heart rate/s; ICU, intensive care unit; IR, impaired relaxation; IVRT, isovolumic relaxation time; LA, left atrium/atrial; LAA, LA appendage; LAP, left atrial pressure; LAV, LA volume; LAVi, LA volume index; LV, left ventricle/ventricular; MAC, mitral annular calcification; MR, mitral regurgitation; MS, mitral stenosis; MV, mitral valve; MVO, mitral valve opening; NL, Nyquist limit; NR, normal range; PASP, pulmonary artery systolic pressure; PN, pseudonormal; PRF, pulse-repetition frequency; PS, pulmonary stenosis; PV, pulmonary vein/s; PW, pulsed-wave; RA, right atrium/atrial; RF, restrictive filling; RV, right ventricle/ventricular; RVOT, RV outflow tract; SFF, systolic filling fraction (= systolic VTI/total forward flow VTI); SR, sinus rhythm; SV, sample volume; TDI, tissue Doppler imaging; TI, time interval; TR, tricuspid regurgitation; US, ultrasound; VTI, velocity time integral; y, year/s; reference values displayed are 5th percentile limit to 95th percentile limit (with 95% confidence limits) (see text and references).

**Table 3:** Main secondary/supplemental echocardiographic parameters for assessing LV diastolic function [1,2,3].

Parameter/Variable	Acquisition	Measurements/Pathophysiological Considerations	Advantages	Limitations
**Valsalva maneuver** **(transmitral inflow)**	- Apical 4C view ± color Doppler imaging. - Preferably, low wall filter setting (100–200 MHz), low signal gain, sweep speed ≤ 50 mm/s, and 10-to-12-second continuous recording during patient’s bearing down against a closed glottis (at rest and during peak strain) are needed. - Adequate maneuver if >10% reduction in maximal E-wave velocity from baseline status.	**Positive:** E/A < 1 or increased A. **Negative:** E/A > 1. Helpful in distinguishing either normal versus PN pattern, or reversible versus irreversible grade 3 diastolic dysfunction (by reducing preload); Highly specific for increased LV FP, during maneuver, if E/A decreases ≥ 50% or A increases (not caused by E-A fusion).	**- If well-performed, good accuracy in diagnosing increased LV FP.**	**- Not every subject can adequately perform the maneuver.**
**Color M-mode Vp**	- Apical 4C view with color Doppler imaging of transmitral inflow (variance mode off). - Alignment optimization of the M-mode cursor with the path of transmitral inflow. - For enhancing the early diastolic slope, color NL lowering is needed.	**Color M-mode Vp (or Vp)** (measured in cm/s): Along the early diastolic slope of first aliased velocity (red–blue interface), measure from the level of mitral annulus to 4 cm into the LV cavity; May be helpful in distinguishing normal versus PN pattern; Vp is indirectly associated with Ƭ (the longer it takes for the LV to relax, the slower the Vp), and E/Vp is directly related to LAP.	**- Relatively load-independent.** **- Reliable index in the presence of LV dysfunction/dilation.**	**- Lower feasibility and reproducibility.** **- In the presence of normal LV volumes/function and high LV FP, possibly misleading normal Vp.**
		**E/Vp ratio (or E/Vp)** (adimensional, after converting E velocity in cm/s). Range: in the presence of reduced LV EF, if ≥2.5 predicts PCWP > 15 mmHg with reasonable accuracy.		
**T_E-e’_-related** **parameters**	For E-wave (transmitral inflow): - Apical 4C view and color/PW Doppler imaging. - Preferably, low wall filter setting (100–200 MHz), low signal gain, PW Doppler SV 1–3 mm, and sweep speed at 100 mm/s are needed. For e’-wave (TDI at mitral annulus): - Apical 4C view with TDI preset optimization *. - Alignment optimization (angle of interrogation should be parallel to annular motion). - Preferably, PW Doppler SV 5–10 mm and sweep speed at 100 mm/s are needed.	**T_E_** (measured in ms): TI between the peak R-wave (on ECG) and the onset of E-wave (transmitral inflow).	**- Generally feasible.** **- May be helpful in diagnosing diastolic dysfunction due to delayed e’ onset compared with E onset (e.g., differentiating RC_prolonged TI_versus PC_usually, not-prolonged TI _).**	**- Non-simultaneous measurements (R-R interval matching is important) in the presence of small TI (thus, increased probability of error).**
		**T_e’_** (measured in ms): TI between the peak R-wave (on ECG) and the onset of e’-wave (TDI).		
		**T_E-e’_** (measured in ms): TI calculated by subtracting T_E_ from T_e’_ (namely, T_e’_—T_E_); May be helpful in distinguishing normal versus PN pattern. In the presence of MS/MR, may be useful for estimating LV FP in combination with IVRT. (**IVRT/T_E-e’_ ratio**; adimensional): in the presence of MS, elevated LV FP if IVRT/T_E-e’_ < 4.2; in the presence of MR, elevated LV FP if IVRT/T_E-e’_ < 5.6.		
**PR ED velocity**	- From any view that allows correct alignment of US beam in parallel with PR jet (CW Doppler). - Preferably, sweep speed at 50–100 mm/s is needed.	**Peak PR ED velocity (or PRED)** (measured in m/s): Measured at ED; Related to PAEDP and PCWP. In the absence of pulmonary disease, PRED ≥ 2 m/s suggests elevated LAP.	**- Generally feasible.** **- Diagnostic and prognostic information.**	**- Misleading in the presence of pulmonary disease.**

Legend: 4C, four-chamber; AF, atrial fibrillation; AFL, atrial flutter; AR, atrial reversal; CAD, coronary artery disease; CW, continuous-wave; DT, deceleration time; ECG, electrocardiogram; ED, end-diastolic; EDP, ED pressure; EF, ejection fraction; FP, filling pressure/s; HCM, hypertrophic cardiomyopathy; IR, impaired relaxation; IVRT, isovolumic relaxation time; LA, left atrium/atrial; LAP, left atrial pressure; LV, left ventricle/ventricular; LVH, left ventricular hypertrophy; MAC, mitral annular calcification; MV, mitral valve; NR, normal range; PA, pulmonary artery; PAEDP, PA ED pressure; PC, pericardial constriction; PCWP, pulmonary capillary wedge pressure; PN, pseudonormal/pseudonormalization; PR, pulmonary regurgitation; PW, pulsed-wave; RC, restrictive cardiomyopathy; RF, restrictive filling; SR, sinus rhythm; SV, sample volume; TDI, tissue Doppler imaging; TI, time interval; US, ultrasound; Vp, early diastolic flow propagation velocity; y, year/s; Ƭ, time constant of LV relaxation; *, low-velocity detection with high-amplitude signals, PW sample volume of 5–10 mm (at septal and lateral insertion site of the mitral leaflets) (see text and references).

**Table 4:** Main advanced echocardiographic techniques for assessing LV diastolic function [1,6,15,16,25].

Parameter/Variable	Acquisition	Measurements/Pathophysiological Considerations	Advantages	Limitations
**LA strain-related parameters**	- 2D apical 4C and 2C views. - Optimize 2D gains, FR (50–70 frames/s), ECG (well-visible P-waves). - Acquire 3-to-5 cardiac cycles for each view to obtain similar HRs.	**LARS** (measured in %): Probe in apical zone with 4C and 2C views; Dedicated (AI-assisted, if available) strain software automatically tracks LA wall (excluding PV and LAA) and calculates LARS; During ventricular systole, peak positive strain value; For assessing diastolic function, LA strain primarily focuses on LARS, directly related to diastolic dysfunction, and inversely related to LV FP. ***- LA strain***: Range (20–39 y): 29.5 (27.6–31.3) to 63.2 (59.9–66.5); Range (40–60 y): 26.8 (25.6–28.0) to 57.7 (55.6–59.9); Range (60–80 y): 24.1 (22.2–26.0) to 52.3 (48.9–55.7). ***- LA strain (TomTec)***: Range (20–39 y): 29.9 (27.0–32.9) to 60.5 (57.6–63.4); Range (40–60 y): 27.5 (25.7–29.4) to 55.4 (53.6–57.2); Range (60–80 y): 25.1 (22.6–27.6) to 50.3 (47.9–52.7). ***- LA strain (EchoPAC)***: Range (20–39 y): 29.5 (27.9–31.1) to 64.9 (59.7–70.2); Range (40–60 y): 25.3 (24.0–26.5) to 61.5 (57.4–65.6); Range (60–80 y): 21.1 (18.7–23.4) to 58.1 (50.3–65.8).	**- Generally feasible and reproducible** (mainly achievable with dedicated software package, adequate operator skills, and a good US window). **- Prognostic and predictive value.**	**- Age-** (LARS decreases with age) **and load-dependent.** **- Challenging or inaccurate in the presence of arrhythmias and other conditions** (e.g., in the presence of BBB, R-R gating may be inaccurate; in the presence of some anatomical conditions, including mobile AS, thin-walled LA, and MAC, LA strain may be inaccurate; in the presence of atrial arrhythmias/stunning, significant MR, HT recipients, preserved EF and GLS > 18%, LARS should not be used to evaluate LV FP). **- Chest-shape-dependent.** **- Image quality-related.** **- Inter-vendor and inter-software variability.**
		**LASCT** (measured in %): Probe in apical zone with 4C and 2C views; Dedicated (AI-assisted, if available) strain software automatically tracks LA wall (excluding PV and LAA) and calculates LASCT as well; Measured in SR as 0 minus strain value at the onset of atrial contraction (namely, pre-A-wave on ECG); Inversely related to LVEDP.		
		**LASCD** (measured in %): Probe in apical zone with 4C and 2C views; Dedicated (AI-assisted, if available) strain software automatically tracks LA wall (excluding PV and LAA) and calculates LASCD as well; Measured in SR as 0 minus strain value at atrial contraction).		
**LV MW**	- 2D apical 4C, 3C, and 2C views. - Optimize 2D gains, FR (40–80 frames/s) and ECG. - Acquire 3-to-5 cardiac cycles for each view to obtain similar HRs. - LV GLS calculation and further derived MW indices (by introducing non-invasive AP and dedicated software package).	**GCW** (MW index measured in mmHg%): positive work evaluated from AVO to AVC and negative work from AVC to MVO.	**- Generally feasible and reproducible** (mainly achievable with dedicated software package, adequate operator skills, and a good US window). **- Prognostic and predictive value.**	**- GLS-related limits** (including image quality). **- AP measurement-related limits.** **- Single available software.**
		**GWI** (MW index measured in mmHg%): total work evaluated from MVC to MVO.		
		**GWE** (MW index measured in %): GCW/(GCW + GWW).		
		**GWW** (MW index measured in mmHg%): positive work evaluated from AVO to AVC and negative work from AVC to MVO.		
**Cardiac THE**	- 2D standard medical ultrasound and continuous external harmonic vibration (patients positioned on a vibration bed). - Vibration bed introduces shear waves into the heart, aiming at creating a mechanical stiffness contrast.	- Regionally resolved mapping of diastolic shear wave (as a myocardial stiffness indicator) is performed (to date, LV posterior wall and interventricular septum have generally been chosen from parasternal long-axis view, for higher reproducibility as well).	**- Diastolic myocardial stiffness detecting and mapping.** **- Accurate at greater depths of up to 15 cm, independently of both region selection and body mass index.** **- Cost-effective technique.**	**- 2D acquisition/evaluation of diastolic phases only.** **- Cardiac THE setup relying on a vibrating bed not easily movable to the patient.**

Legend: 0, generally, negative value, strain value at ED; 2C, two-chamber; 2D, two-dimensional; 3C, three-chamber; 4C, four-chamber; AI, artificial intelligence; AP, arterial pressure; AS, atrial septum; AVC, aortic valve closure; AVO, aortic valve opening; BBB, bundle branch block; BSA, body surface area; ECG, electrocardiogram; ED, end-diastole/diastolic; EDP, end-diastolic pressure; EF, ejection fraction; ES, end-systolic; FP, filling pressure/s; FR, frame rate; GCW, global constructive work; GLS, global longitudinal strain; GWE, global work efficiency; GWI, global work index; GWW, global wasted work; HR/s, heart rate/s; HT, heart transplant; ICU, intensive care unit; IR, impaired relaxation; LA, left atrium/atrial; LAA, LA appendage; LAP, left atrial pressure; LARS, LA reservoir strain; LASCD, LA conduit strain; LASCT, LA contractile/pump strain; LAV, LA volume; LV, left ventricle/ventricular; LVEDP, LV ED pressure; MAC, mitral annular calcification; MR, mitral regurgitation; MV, mitral valve; MVC, mitral valve closure; MVO, mitral valve opening; MW, myocardial work; NR, normal range; PV, pulmonary vein/s; SR, sinus rhythm; THE, time-harmonic elastography; TI, time interval; US, ultrasound; y, year/s; reference values displayed are 5th percentile limit to 95th percentile limit (with 95% confidence limits) (see text and references).

## Data Availability

The data presented in this study are available upon reasonable request from the corresponding author due to privacy.

## References

[1] Nagueh S.F., Sanborn D.Y., Oh J.K., Anderson B., Billick K., Derumeaux G., Klein A., Koulogiannis K., Mitchell C., Shah A. (2025). Recommendations for the evaluation of left ventricular diastolic function by echocardiography and for heart failure with preserved ejection fraction diagnosis: An update from the American Society of Echocardiography. J. Am. Soc. Echocardiogr..

[2] Nagueh S.F., Smiseth O.A., Appleton C.P., Byrd B.F., Dokainish H., Edvardsen T., Flachskampf F.A., Gillebert T.C., Klein A.L., Lancellotti P. (2016). Recommendations for the evaluation of left ventricular diastolic function by echocardiography: An update from the American Society of Echocardiography and the European Association of Cardiovascular Imaging. J. Am. Soc. Echocardiogr..

[3] Nagueh S.F., Appleton C.P., Gillebert T.C., Marino P.N., Oh J.K., Smiseth O.A., Waggoner A.D., Flachskampf F.A., Pellikka P.A., Evangelista A. (2009). Recommendations for the evaluation of left ventricular diastolic function by echocardiography. J. Am. Soc. Echocardiogr..

[4] Mitchell C., Rahko P.S., Blauwet L.A., Canaday B., Finstuen J.A., Foster M.C., Horton K., Ogunyankin K.O., Palma R.A., Velazquez E.J. (2019). Guidelines for performing a comprehensive transthoracic echocardiographic examination in adults: Recommendations from the American Society of Echocardiography. J. Am. Soc. Echocardiogr..

[5] Mor-Avi V., Lang R.M., Badano L.P., Belohlavek M., Cardim N.M., Derumeaux G., Galderisi M., Marwick T., Nagueh S.F., Sengupta P.P. (2011). Current and evolving echocardiographic techniques for the quantitative evaluation of cardiac mechanics: ASE/EAE consensus statement on methodology and indications endorsed by the Japanese Society of Echocardiography. J. Am. Soc. Echocardiogr..

[6] Dell’Angela L., Nicolosi G.L. (2025). Shaping the optimal timing for treatment of isolated asymptomatic severe aortic stenosis with preserved left ventricular ejection fraction: The role of non-invasive diagnostics focused on strain echocardiography and future perspectives. J. Imaging.

[7] Tromp J., Seekings P.J., Hung C.L., Iversen M.B., Frost M.J., Ouwerkerk W., Jiang Z., Eisenhaber F., Goh R.S.M., Zhao H. (2022). Automated interpretation of systolic and diastolic function on the echocardiogram: A multicohort study. Lancet Digit. Health.

[8] Carluccio E., Cameli M., Rossi A., Dini F.L., Biagioli P., Mengoni A., Jacoangeli F., Mandoli G.E., Pastore M.C., Maffeis C. (2023). Left atrial strain in the assessment of diastolic function in heart failure: A machine learning approach. Circ. Cardiovasc. Imaging.

[9] Pandey A., Kagiyama N., Yanamala N., Segar M.W., Cho J.S., Tokodi M., Sengupta P.P. (2021). Deep-learning models for the echocardiographic assessment of diastolic dysfunction. JACC Cardiovasc. Imaging.

[10] Chao C.J., Kato N., Scott C.G., Lopez-Jimenez F., Lin G., Kane G.C., Pellikka P.A. (2022). Unsupervised machine learning for assessment of left ventricular diastolic function and risk stratification. J. Am. Soc. Echocardiogr..

[11] Dell’Angela L., Nicolosi G.L. (2022). Artificial intelligence applied to cardiovascular imaging, a critical focus on echocardiography: The point-of-view from “the other side of the coin”. J. Clin. Ultrasound.

[12] Dell’Angela L., Nicolosi G.L. (2022). Artificial intelligence in clinical echocardiography: Many expectations, but deep uncertainties for defining strategies to overcome difficulties and obstacles. J. Am. Soc. Echocardiogr..

[13] Tseng A.S., Lopez-Jimenez F., Pellikka P.A. (2022). Artificial intelligence in clinical echocardiography: Many expectations, but deep uncertainties for defining strategies to overcome difficulties and obstacles: Authors’ reply. J. Am. Soc. Echocardiogr..

[14] Brutsaert D.L., Sys S.U., Gillebert T.C. (1993). Diastolic failure: Pathophysiology and therapeutic implications. J. Am. Coll. Cardiol..

[15] Mihos C.G., Liu J.E., Anderson K.M., Pernetz M.A., O’dRiscoll J.M., Aurigemma G.P., Ujueta F., Wessly P., on behalf of the American Heart Association Council on Peripheral Vascular Disease, Council on Cardiovascular and Stroke Nursing (2025). Speckle-tracking strain echocardiography for the assessment of left ventricular structure and function: A scientific statement from the American Heart Association. Circulation.

[16] Thomas J.D., Edvardsen T., Abraham T., Appadurai V., Badano L., Banchs J., Cho G.Y., Cosyns B., Delgado V., Donal E. (2025). Clinical applications of strain echocardiography: A clinical consensus statement from the American Society of Echocardiography developed in collaboration with the European Association of Cardiovascular Imaging of the European Society of Cardiology. J. Am. Soc. Echocardiogr..

[17] Di Virgilio E., Monitillo F., Santoro D., D’alessandro S., Guglielmo M., Baggiano A., Fusini L., Memeo R., Rabbat M.G., Favale S. (2021). Mid-diastolic events (L events): A critical review. J. Clin. Med..

[18] Fortuni F., Biagioli P., Myagmardorj R., Mengoni A., Chua A.P., Zuchi C., Sforna S., Bax J., Marsan N.A., Ambrosio G. (2024). Left atrioventricular coupling index: A novel diastolic parameter to refine prognosis in heart failure. J. Am. Soc. Echocardiogr..

[19] Wang Y.H., Dong Y., Li G.Y., Ma C.-Y. (2025). Unveiling the left atrioventricular coupling index: A promising marker for diastolic dysfunction and prognosis. J. Am. Soc. Echocardiogr..

[20] Fortuni F., Biagioli P., Carluccio E. (2025). Reply to unveiling the left atrioventricular coupling index: A promising marker for diastolic dysfunction and prognosis. J. Am. Soc. Echocardiogr..

[21] Zornitzki L., Topilsky Y. (2024). Left atrioventricular coupling index: When minimal left atrial volume is actually ‘more’ than maximal left atrial volume. J. Am. Soc. Echocardiogr..

[22] Anwar A.M., Alshammakh M.S., Eyaz M., Al-Katheri A., Abdelfattah A.N., Ali M.A.M., Albakri I. (2025). Normal reference values of left atrioventricular coupling index on two-dimensional echocardiography. Int. J. Cardiovasc. Imaging.

[23] Tran H.M., Truong H.P., Tran C.C., Vo T.M., Nguyen D.N.Q., Dao L.T. (2025). Analysis of factors related to early left ventricular dysfunction in hypertensive patients with preserved ejection fraction using speckle tracking echocardiography: A cross-sectional study in Vietnam. Diagnostics.

[24] Sonaglioni A., Nicolosi G.L., Trevisan R., Lombardo M., Grasso E., Gensini G.F., Ambrosio G. (2023). The influence of pectus excavatum on cardiac kinetics and function in otherwise healthy individuals: A systematic review. Int. J. Cardiol..

[25] Meyer T., Wellge B., Barzen G., Chandia S.K., Knebel F., Hahn K., Elgeti T., Fischer T., Braun J., Tzschätzsch H. (2025). Cardiac elastography with external vibration for quantification of diastolic myocardial stiffness. J. Am. Soc. Echocardiogr..

[26] Rodriguez-Sanchez I., Villanueva-Benito I., Agirre U., Onaindia J.J., Urkullu A., Cacicedo A., Ullate A., Bravo I., Florido J., Salcedo A. (2025). Diastolic function and cardiovascular events in patients with preserved left ventricular ejection fraction. Improving risk stratification with left atrial strain. Front. Cardiovasc. Med..

[27] Tolvaj M., Zhubi Bakija F., Fábián A., Ferencz A., Lakatos B., Ladányi Z., Szijártó Á., Edvi B., Kiss L., Szelid Z. (2025). Integrating left atrial reservoir strain into the first-line assessment of diastolic function: Prognostic implications in a community-based cohort with normal left ventricular systolic function. J. Am. Soc. Echocardiogr..

[28] Smiseth O.A., Aalen J.M. (2025). Imaging of left ventricular diastolic function: Do we need both left atrial volume and reservoir strain?. J. Am. Soc. Echocardiogr..

[29] Elhady F., Ali A.A., Elkholy N.S., El-Mnakhly E.A., Elkareem T.S.A., Galal A., Negm M.A. (2025). Left atrial filling index and stiffness index and its correlation to the duration of diabetes in patients with type II diabetes mellitus. Int. J. Cardiovasc. Imaging.

[30] Liang H., Zhao X., Xiao C., Sun L., Zhang F. (2025). Four-dimensional echocardiographic quantification of left atrial function in metabolic syndrome across different diastolic function states. BMC Cardiovasc. Disord..

[31] Mannina C., Ito K., Jin Z., Yoshida Y., Russo C., Nakanishi K., Rundek T., Homma S., Elkind M.S., Di Tullio M.R. (2025). Left atrial function and incident heart failure in older adults. J. Am. Soc. Echocardiogr..

[32] Gegenava T., Nieman K. (2025). Left atrial volumetric/mechanical coupling index: Best of both worlds?. J. Am. Soc. Echocardiogr..

[33] Dell’Angela L., Nicolosi G.L. (2024). From ejection fraction, to myocardial strain, and myocardial work in echocardiography: Clinical impact and controversies. Echocardiography.

[34] Chilingaryan A., Tunyan L., Arzumanyan M., Balyan H. (2025). Predictive value of left ventricular myocardial constructive work in patients with heart failure with preserved ejection fraction and preclinical diastolic dysfunction. J. Cardiovasc. Imaging.

[35] Sokratous S., Kyriakou M., Khattab E., Alexandraki A., Fotiou E.L., Chrysanthou N., Papakyriakopoulou P., Korakianitis I., Constantinidou A., Kadoglou N.P.E. (2025). The role of diastolic stress echo and myocardial work in early detection of cardiac dysfunction in women with breast cancer undergoing chemotherapy. Biomedicines.

[36] Jasaityte R., Bajraktarevic R., Blaschke-Waluga D., Seeland U., Regitz-Zagrosek V., Landmesser U., Stangl K., Knebel F., Stangl V., Brand A. (2023). Determinants of myocardial work indices in women. Echocardiography.

[37] Grapsa J., Argulian E., Smiseth O.A. (2025). Diastolic dysfunction: A comparison of 2025 ASE, 2024 BSE and 2022 EACVI guidelines. Eur. Heart J. Cardiovasc. Imaging.

[38] Taub C.C., Stainback R.F., Abraham T., Forsha D., Garcia-Sayan E., Hill J.C., Hung J., Mitchell C., Rigolin V.H., Sachdev V. (2025). Guidelines for the standardization of adult echocardiography reporting: Recommendations from the American Society of Echocardiography. J. Am. Soc. Echocardiogr..

[39] Dell’Angela L., Sonaglioni A., Nicolosi G.L. (2025). Performing and reporting duration of the optimal echocardiographic exam in current clinical practice: Time to turn the page?. J. Am. Soc. Echocardiogr..

[40] Stainback R.F., Taub C.C. (2025). Reply to: Performing and reporting duration of the optimal echocardiographic exam in current clinical practice: Time to turn the page?. J. Am. Soc. Echocardiogr..

